# Infant EEG activity as a biomarker for autism: a promising approach or a false promise?

**DOI:** 10.1186/1741-7015-9-61

**Published:** 2011-05-20

**Authors:** Richard Griffin, Chris Westbury

**Affiliations:** 1 Department of Psychology & Center for Cognitive Studies, Tufts University, Medford, MA, USA; 2 Department of Psychology, University of Alberta, Edmonton, AB, Canada

## Abstract

The ability to determine an infant's likelihood of developing autism via a relatively simple neurological measure would constitute an important scientific breakthrough. In their recent publication in this journal, Bosl and colleagues claim that a measure of EEG complexity can be used to detect, with very high accuracy, infants at high risk for autism (HRA). On the surface, this appears to be that very scientific breakthrough and as such the paper has received widespread media attention. But a close look at how these high accuracy rates were derived tells a very different story. This stems from a conflation between "high risk" as a population-level property and "high risk" as a property of an individual. We describe the approach of Bosl *et al*. and examine their results with respect to baseline prevalence rates, the inclusion of which is necessary to distinguish infants with a biological risk of autism from typically developing infants with a sibling with autism. This is an important distinction that should not be overlooked.

Please see research article: http://www.biomedcentral.com/1741-7015/9/18 and correspondence article: http://www.biomedcentral.com/1741-7015/9/60

## Introduction

In some ways, scientists investigating early autism (ASD) face similar problems to those investigating climate change: by the time that we are certain of our results, it may be too late to do anything about it. Good science, like good medicine, should be predictive and preventative. It is not possible to diagnose autism in early infancy because it is defined by behavioral criteria that are not manifest until after the first or second birthday (for example, language impairments). Given the goal of prevention, it is necessary to study children at ages too young for clinical presentation. This will entail the study of infants and the development of measures that do not rely solely on overt behavior. This is precisely the approach taken by Bosl and colleagues [1] in their recent publication in this journal, entitled "EEG complexity as a biomarker for autism spectrum disorder risk."

## Discussion

Due to the potential importance of this research, the paper has received press attention from all the major news outlets, and will likely receive more. But the design of the study is novel and complicated and the results are hard to interpret, even for researchers in the field. Moreover, the findings, broadly construed, are all too easy to misinterpret and naturally lead to the false conclusion that scientists have discovered a technique to detect autism in infancy using EEG. While the authors do not claim this, many news outlets do and the headline "Novel biomarker for Autism Spectrum Disorder?" comes from this very journal. A close look at the study gives a very clear answer: No, we have not discovered a biomarker for ASD - not yet, at least.

The central claim of their paper is not that EEG can be used to detect a potential biomarker for autism; it is that EEG can be used to detect a potential biomarker for infants at "high risk" for autism (HRA). The implications of the study hinge on what is meant by HRA. A commonsense interpretation would lead one to believe that the infants in this study were identified as HRA based on their neurological activity. But HRA, as it is used in Bosl *et al*., is a technical term that refers to the relative risk of a given population or demographic. In this case, HRA refers specifically to "infant siblings of child with autism". This population is important to study because epidemiological research has found higher rates of ASD as compared to the population as a whole [2-4]. This increased risk thus improves researchers' chances of studying a child who will later be diagnosed with autism.

Of course, we do not need an EEG to identify these siblings because we already know who they are. What we want to know is whether EEG can be used to predict whether a specific child will develop autism. That is, we want to move from HRA at the population level to HRA at the individual/biological level. To this end, we can use current prevalence (that is, risk) estimates to predict how many children in a given sample will be diagnosed with ASD.

The prevalence of ASD in the general population is around 1 in 100 or 1%. The prevalence (relative risk) is higher in siblings of a child with autism (around 5%) and is even higher in later-born siblings. This is known as recurrence risk and it increases to around 10% [2-4]. These later-born siblings constitute the HRA group in Bosl *et al*.'s recent publication. "High risk" may be an appropriate label if we consider that the relative risk of this population is 10 times higher than that of the comparison group (CON) in this study. Put another way, finding 10 pre-ASD infants from a random sample of the general population would require a sample size of around 1,000. Finding the same number using an HRA group would only require a sample size of roughly 100, making this a highly sought after population for autism research.

The media-friendly finding provided by Bosl and colleagues was that a non-invasive neurological measure could be used to distinguish between HRA infants and non-HRA infants with around 80% accuracy, and sometimes higher. Accuracy for boys at nine months of age was close to 100%. This sounds impressive. But risk, as explained above, is a population-level property; it cannot also be an internal property of all or even most of the individuals in that population. Groups are not diagnosed with autism - people are. It appears that Bosl *et al*. have conflated this important distinction and a closer look at the study reveals how this was done.

The paper, at its core, is not about autism. It is about whether a technique for deriving the relative complexities of EEG signals can be used in what is essentially a two-category (HRA or CON) sorting task. The technique creates a measure of multiscale entropy (ME). The ME profiles are the data (dependent measures) that will require sorting. The task of sorting was carried out by three statistical learning techniques, the most successful of which was a Bayesian classification algorithm. Via supervised learning (that is, learning from its mistakes), the program learned to correctly sort the ME data into the two categories quite well, averaging around 80% accuracy, and sometimes higher, as noted above.

The accuracy of the learning algorithms could be determined because the researchers knew with 100% accuracy which child had a sibling with ASD (HRA) and which child did not (CON). This is also how the learning algorithms were able to learn from their mistakes. The correct answers were already known. It remains to be seen whether ME measures can tell us about actual/biological risk for autism, but it is clear that the learning algorithms were trained with ME data that will not predict autism the large majority of the time. In fact, by and large, the higher the accuracy rates derived from these ME profiles, the less likely that those MEs will be biomarkers for ASD.

This is best illustrated by keeping relative risk at the population level, where it belongs. Thus, even though ASD risk is 10 times higher for the HRA group, it is still the case that roughly 90% of the infants in the Bosl *et al*. study will not receive a diagnosis of ASD [2-4]. Given this, a report of 100% accuracy will include MEs that will not lead to ASD in 90% of those tested; that is, if used as diagnostic for ASD, those MEs would produce a false positive or false alarm rate of 90%. Only when we incorporate baseline prevalence rates (that is, the likelihood of having the condition prior to testing) can we determine a diagnostic test's positive predictive value (PPV) [5]. This can be done via Bayes' Rule [6], which Bosl *et al*. did not include. We want to know the PPV because it tells us the proportion of individuals with a positive test that actually has (or will have) the condition. Using the MEs that were correctly sorted 80% of the time will result in a positive predictive value of around .30, or 30%. A diagnostic test that promises to be wrong most of the time would likely do more harm than good. (The precise value will vary by condition. Using the data from the 18-month-olds, which reported a specificity of .8, we calculated a PPV of .307. The calculation using Bayes' Rule is as follows:

Note that this calculation assumes that the test is only given to siblings of those diagnosed with ASD. If the test were used in a general population, the base rate for ASD would be much lower and the test's utility at identifying those who might have ASD would be much worse. For example, the PPV would be 0.04 if we assumed a base rate of 0.01.)

Nevertheless, research with these siblings is crucial to our understanding of the neurological underpinnings of autism, and the data from Bosl *et al*. may turn out to be groundbreaking, albeit not in its present form. In addition to the increased risk of ASD, 10 to 20% of later-born siblings may show some autism-like symptoms (for example, language delay) without meeting formal criteria, and family members of an individual with ASD have higher rates of psychiatric and developmental disorders compared to the general population [2,3]. As suggested by the authors, ME may index an endophenotype, that is, some shared factor or trait indicative of specific (shared) genetic contributions to a disease or disorder. This may be the case, but twin studies point to largely non-overlapping genetic influences on the various features of autism [7] and an increasing number of cases of ASD are associated with *de novo *mutations [8]. Increased ME might also reflect epigenetic effects. For instance, infant EEG activity is known to be influenced by maternal depression [9] and mothers of children with developmental disabilities show increased rates of depression and anxiety [10]. Whatever the case, abnormal brain development is the proximate cause of ASD and Bosl *et al*.'s data are important regardless of whether a specific genetic contribution is isolable.

## Conclusions

While it may not be scientifically interesting to identify all or even most of these siblings via the same neurological marker, we know that some of these children will meet formal criteria for ASD and others will show related anomalies. By tracking the development of these children, their various outcomes can then be used to look back at the data presented here. Once these outcomes are known, the ME profiles of these specific children, at various stages of development, will become the measures of keen interest and may well represent a biomarker for ASD. These ME profiles could even be used to help the learning algorithms find ME profiles that are likely to lead to ASD or a milder impairment, or those that are predictive of a typically developing child. This will be very important information indeed, and such a finding would be worthy of widespread media attention. The discovery of such a biomarker will, however, render the present results of the learning procedure's accuracy almost useless. Rather than lumping the siblings into a single group, we want to know what is different about them. Bosl *et al*. have the data that could very well tell us what those differences are and we very much look forward to their follow-up work. Indeed, using the baseline prevalence rates discussed above as a constraint, a different analysis of their current data should be able to generate predictions as to which individuals are likely to develop atypically and which are not, even before these individual outcomes are known. Once we are able to distinguish between "high risk" at the population level from "high risk" at the biological/individual level, we will have the kind of predictive tools best suited for the ultimate aim of prevention.

## Abbreviations

ASD: Autism Spectrum Disorder; CON: control/comparison group; HRA: high risk for autism;

## Competing interests

The authors declare that they have no competing interests.

## Authors' contributions

RG and CW contributed equally to the content of this article. Both authors have read and approved the final manuscript

## Pre-publication history

The pre-publication history for this paper can be accessed here:

http://www.biomedcentral.com/1741-7015/9/61/prepub
